# Further Assessment of Monkeypox Virus Infection in Gambian Pouched Rats (*Cricetomys gambianus*) Using In Vivo Bioluminescent Imaging

**DOI:** 10.1371/journal.pntd.0004130

**Published:** 2015-10-30

**Authors:** Elizabeth A. Falendysz, Juan G. Lopera, Faye Lorenzsonn, Johanna S. Salzer, Christina L. Hutson, Jeffrey Doty, Nadia Gallardo-Romero, Darin S. Carroll, Jorge E. Osorio, Tonie E. Rocke

**Affiliations:** 1 U.S. Geological Survey-National Wildlife Health Center, Madison, Wisconsin, United States of America; 2 Department of Pathobiological Science, School of Veterinary Medicine, University of Wisconsin, Madison, Wisconsin, United States of America; 3 Centers for Disease Control and Prevention, National Centers for Zoonotic and Vector-Borne and Enteric Diseases, Division of High Consequence Pathogens and Pathology, Poxvirus and Rabies Branch, Atlanta, Georgia, United States of America; Stanford University School of Medicine, UNITED STATES

## Abstract

Monkeypox is a zoonosis clinically similar to smallpox in humans. Recent evidence has shown a potential risk of increased incidence in central Africa. Despite attempts to isolate the virus from wild rodents and other small mammals, no reservoir host has been identified. In 2003, *Monkeypox virus* (MPXV) was accidentally introduced into the U.S. via the pet trade and was associated with the Gambian pouched rat (*Cricetomys gambianus*). Therefore, we investigated the potential reservoir competence of the Gambian pouched rat for MPXV by utilizing a combination of in vivo and in vitro methods. We inoculated three animals by the intradermal route and three animals by the intranasal route, with one mock-infected control for each route. Bioluminescent imaging (BLI) was used to track replicating virus in infected animals and virological assays (e.g. real time PCR, cell culture) were used to determine viral load in blood, urine, ocular, nasal, oral, and rectal swabs. Intradermal inoculation resulted in clinical signs of monkeypox infection in two of three animals. One severely ill animal was euthanized and the other affected animal recovered. In contrast, intranasal inoculation resulted in subclinical infection in all three animals. All animals, regardless of apparent or inapparent infection, shed virus in oral and nasal secretions. Additionally, BLI identified viral replication in the skin without grossly visible lesions. These results suggest that Gambian pouched rats may play an important role in transmission of the virus to humans, as they are hunted for consumption and it is possible for MPXV-infected pouched rats to shed infectious virus without displaying overt clinical signs.

## Author Summary

*Monkeypox virus* is a close relative of *Variola virus*, the agent of smallpox, and causes a similar disease in humans, with classic pox skin lesions. Up to 10% mortality has been associated with some strains of monkeypox. Monkeypox disease occurs in central and west Africa. It is transmitted to humans from wild animals, but the reservoir species that maintains this virus in nature has not been identified. The Gambian pouched rat (*Cricetomys gambianus*) was implicated as a possible source during the 2003 US monkeypox outbreak which resulted in human disease. To understand how the Gambian pouched rat could maintain and transmit the virus, six Gambian pouched rats were infected with *Monkeypox virus* engineered to express a marker gene, luciferase, which is detectable within the live animal via bioluminescence. Two animals served as uninfected controls. Viral shedding was quantified. Two of three animals infected via the intradermal route became clinically ill. Those infected intranasally showed no signs of disease, but the virus was detected in the nasal and oral cavities and distant sites within their bodies. All infected animals shed live *Monkeypox virus*. This study indicates that Gambian pouched rats could serve to transmit *Monkeypox virus* to humans in Africa.

## Introduction

Monkeypox (MPX) is an emerging disease caused by an Orthopoxvirus, *Monkeypox virus* (MPXV). It is closely related to smallpox, although it has a much lower mortality rate in humans, around 10% for the Congo Basin clade of MPX, compared to up to 40% mortality for smallpox [1,2]. Unlike smallpox, MPX is a zoonosis. Historically, MPXV has circulated in central and west Africa but was first discovered in 1958 in imported captive monkeys in Copenhagen [3]. The prevalence and distribution of this disease first became of interest in the 1970s during the smallpox eradication campaign, as public health agencies needed a way to differentiate between cases of MPX and smallpox. Analysis of human clinical samples revealed that there are two geographically distinct clades of the virus, west African and central African [4]. In west Africa, no human cases of MPX have been reported since the 1980s, although serological studies have demonstrated that anti-orthopox antibodies are still present in humans from this region [5,6]. In contrast, the number of cases of MPX continues to increase in central Africa [7]. Historically the majority of MPX cases have been reported in Democratic Republic of Congo (DRC), but newer reports of MPX have emerged from the neighboring countries of Republic of Congo (ROC) and Sudan [1,8]. The reasons for the change in incidence and geographic distribution of MPX are unclear. Identification of the reservoir host(s) is necessary to build a more thorough understanding of the epidemiology of Monkeypox in Africa and to develop prevention strategies to reduce new human cases.

Despite several attempts to identity the wildlife reservoir host during the smallpox eradication campaign, MPXV was isolated only once from a moribund rope squirrel (*Funisciurus anerythrus*) in Zaire (now Democratic Republic of Congo)[9]. Recently, Monkeypox was isolated from a juvenile sooty mangabey (*Cercocebus atys*) in Côte d’Ivoire[10]. Additionally, serological evidence of infection with MPXV has been documented in several species, including the Gambian pouched rat (*Cricetomys gambianus)*, rope squirrels (*Funisciurus spp*.), sun squirrels (*Heliosciurus spp*.), African dormice (*Graphiurus kelleni*), multimammate mice (*Mastomys natalensis*) and tiny fat mice (*Steatomys parvus)* [6,11,12,13]. Orthopoxvirus DNA has been detected by PCR in pouched rats, African dormice, and African ground squirrels (*Xerus spp*.)[14,15]. Although *MPXV* has been found in primates, evidence of infection has been found more commonly in rodents [16]. Both terrestrial rodents and squirrels are utilized as a food source in Central Africa [17]. The pouched rat is a commonly eaten species, as it is large and relatively easy to hunt [18]. For this reason, Gambian pouched rats are of special interest as a potential reservoir host.

In vivo imaging has been used extensively to detect fluorescent or luminescent signals in live animals. These signals can be coupled with pathogens, antibodies, or cancer cells to study the distribution of cells, antigens, or pathogens inside the live animal through the duration of infection. This technique was originally developed for mice and has been used to study various bacterial, viral, and protozoan pathogens [19,20,21,22,23]. A major advantage of this technique is that individual animals can be tracked through time, rather than sacrificing many animals at various time points throughout the study.

Bioluminescent imaging (BLI) specifically refers to in vivo imaging using luminescent signals. The luminescent signals are quantifiable and previous studies have demonstrated that luminescence correlates with pathogen load in vitro [19,22]. Studies in mice, prairie dogs, and dormice have shown that dissemination of recombinant MPXV expressing luciferase can be tracked between and within internal organs and can be quantified to compare relative viral load [24,25,26,27]. In the case of viral pathogens, such as MPXV, luminescence is produced as the virus is replicating and producing the luciferase enzyme, therefore luminescence is used as an indicator of viral replication. BLI is a useful technique to track the distribution and shedding of MPXV in potential reservoir hosts, especially when the number of individuals captured is low and there may be large amounts of variation in outbred, wild animals. We used BLI to assess reservoir competence for MPXV in Gambian pouched rats as a part of a larger effort to determine which species may maintain MXPV in nature and their epidemiological relationship to human infection.

## Materials and Methods

### Ethic Statement

This work was approved by the Animal Care and Use Committee of the National Wildlife Health Center, protocol number EP090616A6. Anesthesia was performed using injectable medetomidine and isoflurane inhalant anesthetic. Euthanasia was performed by CO_2_ asphyxiation.

### Animals

An MPXV challenge study was conducted in eight Gambian pouched rats (*Cricetomys gambianus*). Gambian pouched rats were wild-caught using Tomahawk live traps (Tomahawk Live Trap Co, Tomahawk, WI, USA) in Grassy Key, Florida, USA. Trapping was conducted in collaboration with the Florida Fish and Wildlife Conservation Commission and the U.S. Department of Agriculture. Pouched rats have become an invasive species in southern Florida after eight animals escaped from a breeder for the pet trade in approximately 1999 [28,29]. These animals have continued to breed and live in the Florida Keys [29] and have the potential to spread across large areas of North America [30]. One animal (GPR 14) was caught as a juvenile and inoculated in this study as a young adult, while all other animals in this study were adults when captured and inoculated. They were treated with a topical parasiticide (Revolution, Zoetis (formerly Pfizer Animal Health), Florham Park, NJ, USA) and transferred to the USGS National Wildlife Health Center (NWHC). After 60 days of quarantine, they were treated again with topical parasiticide and separated into groups based upon their trapping dates. Pouched rats were housed in 5 square foot individual metal cages with stainless steel drop-through floors (Multi-species Modular caging, Lab Products, Inc. Seaford, DE, USA). Cages were placed in a cage rack, similar to standard laboratory cat housing. They were given several sheets of paper pan liner (Harlan, Indianapolis, IN, USA) and large PVC pipes or rat boxes inside their cages to use as nesting materials. Rats were fed a 50/50 mixture of commercial laboratory rodent block and dog food (Teklad Global #2019, and #2021, Harlan, Indianapolis, IN, USA), and water *ad libitum*. They also received 1–2 pieces of apple, sweet potato or carrot daily. The room temperature ranged from 68 to 70° F, with a 12h:12h light cycle. Upon initiation of the study, weights ranged from 950 to 1150 g.

### Experimental Infection

For this challenge study, we used central African MPXV (ROC 2003–358) expressing firefly luciferase (MPXV/Luc+). The production and characteristics of this virus have been described elsewhere [24]. In previous in vivo and in vitro studies, this recombinant virus did not differ in virulence compared to its parental MPXV strain. Three pouched rats, two adult males and one adult female, were infected intradermally (ID) with 10^6^ plaque forming units (pfu) of MPXV/Luc+. A 1 inch square area of skin was shaved on the intrascapular area of the dorsum; 10 ul of PBS containing the virus was placed on this shaved area of skin and a 25 gauge needle was used to pierce the skin 10 times through the droplet, similar to Hutson *et*. *al*. [31]. One adult male animal was mock-infected with PBS using the same method and was housed in a separate room, to be used as a negative control. Three additional pouched rats (one adult male, one adult female, and one young adult male) were infected intranasally (IN), using 5 ul of PBS containing 0.5 x 10^6^ pfu in each nostril, for a total dose of 1 x 10^6^ pfu in 10ul. One adult female animal was mock-infected with PBS in the same manner. This animal was housed in the same room, to be used as a sentinel for aerosol transmission. All animals were anesthetized before infection, using intramuscular medetomidine (Dormitor, Novartis, Basel, Switzerland) as a premedication, followed by isoflurane (Phoenix Pharmaceutical, St. Joseph, MO, USA) for induction and maintenance of anesthesia. Medetomidine was reversed for recovery using an equal volume of intramuscular atipamazole (Antisedan, Zoetis, Florham Park, NJ, USA). Premedication was used to reduce the amount of isoflurane necessary and to decrease the likelihood that an animal would arouse from anesthesia during imaging and pose a risk to the person handling them. Following infection, animals were observed daily for signs of illness and were euthanized if they displayed difficulty in eating, drinking, or breathing, or if they displayed greater than 20% weight loss. Euthanasia was performed using CO_2_ asphyxiation.

### In Vivo Bioluminescent Imaging and Sampling

Prior to sampling and BLI, all animals were anesthetized as described above. BLI was performed on 1, 4, 7, 11, 14, 18, 21, 25, 28, and 34 days post inoculation (dpi) for the ID infected group and 1, 4, 8, 12, 15, 19, 22, and 26 dpi for the IN infected group. The negative control animal was anesthetized for blood and swab collection on 1, 7, 14, 21, and 31 dpi, but was only imaged once, prior to mock infection, to reduce the possibility of inadvertent infection. This image was used to estimate the background level of luminescence in pouched rats. The sentinel animal was imaged and sampled on 8, 15, 22 and 26 dpi. When the sentinel was imaged and sampled, this was performed before all other animals and all equipment was disinfected with Maxima 256 detergent (Brulin, Indianapolis, IN, USA) followed by 70% ethanol between each animal. For imaging, 125 mg/kg of D-luciferin potassium salt (GoldBio, St. Louis, MO, USA) in Dubelco’s PBS was injected intraperitoneally, after induction of anesthesia. The animals were imaged in four views, beginning 45 minutes after injection of luciferin. Dorsal, ventral, and two lateral images were acquired using an IVIS 200 series in vivo imager (Caliper Life Science, Alameda, CA, USA). Images were collected and analyzed using Living Image 4.2 (Caliper Life Science, Alameda, CA, USA). Region of interest (ROI) analysis was performed using hand drawn ROIs that encompassed the portion of the animal that was in the viewing area. Adult pouched rats are slightly longer than the largest viewing dimensions of the imager, but by imaging in 4 views, all areas of the animals were analyzed. Total luminescence was estimated by adding luminescence (p/s/cm^2^/sr) from dorsal and ventral views. Lateral images did not identify luminescence that was not detectable by either dorsal or ventral views, and so further analysis was not completed for these images.

While under anesthesia, animals were also weighed, examined for lesions, bled, and swab and urine samples were collected. Sterile polyester swabs were used to sample oral and nasal cavities and rectum. Pre-moistened swabs were used to sample the corneal and conjunctival surfaces of both eyes. All swabs were placed into tubes containing 400 ul of DMEM supplemented with 1 μg/L amphotericin B, 100 U/ml penicillin, 100 μg/ml streptomycin, and 0.05 mg/ml gentamycin (Life Technologies, Grand Island, NY, USA). Blood was collected from the tail vein and placed into EDTA and serum separator tubes. If the bladder was palpable during sample collection, urine was manually expressed into a sterile cryovial. All samples were refrigerated at 4°C until they could be frozen at -80°C after imaging. Serum separator tubes were centrifuged at 2000 rpm for 10 minutes before being frozen at -80°C.

### Necropsy and Sample Processing

Animals were necropsied within 24 hours of death. Animals that were not necropsied immediately were refrigerated at 4°C until necropsy. A minimum of 13 tissues were collected: brain, submandibular lymph node, salivary gland, heart, lung, skin (including lesions, if present), stomach, intestine (several pieces including duodenum, jejunum and ileum), liver, spleen, kidney, and either testis or ovary. Immediately after necropsy, tissues were frozen at -80°C for later processing. Tissue pieces (40–100 mg) were homogenized using the Bullet Blender Storm bead homogenizer (Next Advance, Averill Park, NY, USA) according to tissue-specific protocols [32]. PBS with 1% FBS was added to tissue slurries to make a 10% solution by weight. DNA was extracted from 200 μl of tissue slurry, whole blood or swab liquid, using the QiaAMP DNA mini kit (Qiagen, Hilden, Germany). For a small number of tissue samples, PCR inhibitors remained in the DNA sample after extraction. The DNA extractions were repeated with Zymo tissue g-DNA kit (Zymo Research, Irvine, CA). DNA was extracted from urine using standard phenol-chloroform extraction followed by ethanol precipitation [33].

### Real Time PCR

Viral DNA was detected in blood and tissues using real time PCR to detect the E9L gene of European orthopox viruses as described by Li *et al* [34] with minor modifications: Taqman Universal Master Mix (Applied Biosystems, Foster City, CA, USA) was used in the DNA Engine Opticon (Bio-Rad, Hercules, CA, USA). Standards of MPXV DNA (Congo strain) ranging from 54 to 5.4 x 10^−5^ ng were used as positive controls and to determine the sensitivity of each assay. The cutoff was chosen as any fluorescence value two standard deviations above background measured during cycles 3–10. Samples that surpassed the cutoff by 40 cycles were considered positive. All assays were sensitive enough to detect 5.4 x 10^−4^ ng of MPXV DNA, approximately 2600 viral genomes.

### Viral Titration

Virus in swabs and tissue homogenates was titered by TCID_50_ plate titration. Homogenates and swab media were thawed and serially diluted by a factor of 10 from 10^−1^ to 10^−5^ or 10^−10^. Each dilution (100 μl) was incubated in 8 wells each of a 96 well plate with Vero cells (ATCC CCL-81, Manassas, VA, USA) grown to 90% confluency. Plates were incubated for 3 days at 37°C, 5% CO_2_ before cells were fixed using 1.5% crystal violet in 10% buffered formalin. Any wells with cytopathic plaques were considered positive and viral titer was calculated using a Reed and Muench calculator [35]. Mean shedding per day at each anatomic site (ocular, oral, nasal, and rectal swabs) was calculated using GraphPad Prism software (GraphPad Software Inc., La Jolla, CA, USA). Each animal’s daily shedding was added to calculate the total shedding per individual, and a mean total shedding was calculated for each route of inoculation. Unpaired *t* tests with Welch’s correction (α = 1%) were performed to compare the mean total shedding for each site between inoculation routes.

### ELISA

Antibodies were detected using an ELISA as described in Hutson et al [31]. Briefly, each plate was coated with lysate from vaccinia-infected Vero cells on one half and uninfected cell lysate on the other. Samples were plated from 1:50 to 1:1600 dilutions on both sides of the plate and all were run in duplicate. The secondary antibody was a protein A/G conjugate, followed by SureBlue peroxidase substrate (KPL #52-00-01, Kirkegaard and Perry Laboratories, Washington DC, USA). Plates were read at 450nm in a plate reader. Controls were sera from a vaccinia-immune human, an uninfected mouse, and the negative control pouched rat (GPR 51). The optical density (OD) of the entire lysate half of the plate was averaged. Any sample that had an average OD two standard deviations greater than the average of the lysate wells was considered positive.

## Results

### Bioluminescent Imaging

**Fig 1:**
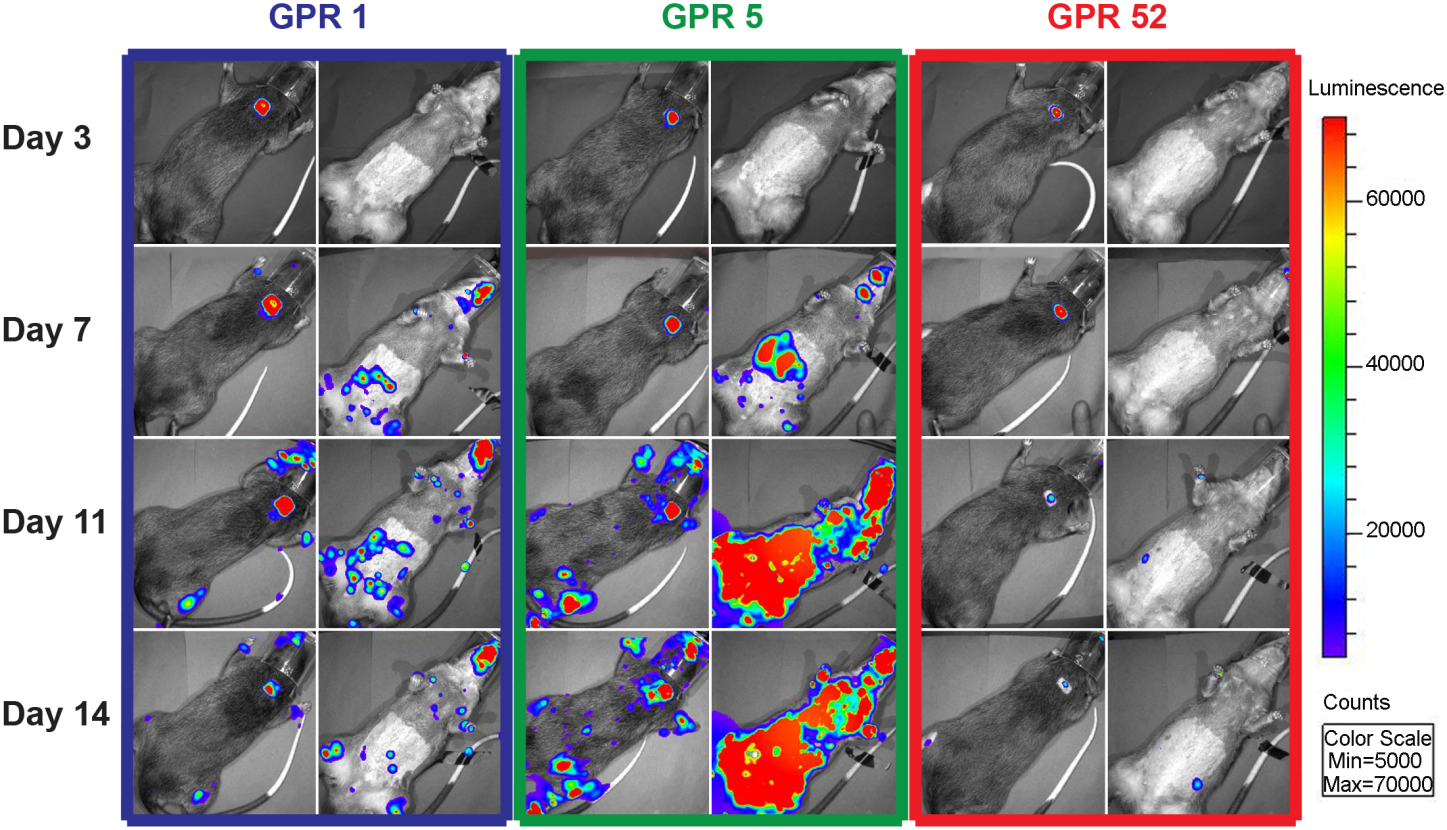
Dorsal and ventral views of Gambian pouched rats (*Cricetomys gambianus*) infected intradermally with MPXV/Luc+. Gambian pouched rats that were infected intradermally with *Monkeypox virus* that expresses firefly luciferase (MPXV/Luc+) show luminescence, indicative of virus replication, at the initial site of inoculation, over the dorsal scapulae, and then at distant sites. The intensity of light produced is used to estimate the quantity of replicating MPXV/Luc+. By day 7, luminescence is visible in the oronasal area of all three infected rats. This is corroborated by tests for viral shedding; all three animals had detectable virus in oral and nasal swabs. Viral replication continues in GPR1, especially in the location of numerous abdominal skin lesions. GPR 5 developed systemic infection, with a large amount of luminescence detected in the abdominal and thoracic regions by day 14.

**Fig 2:**
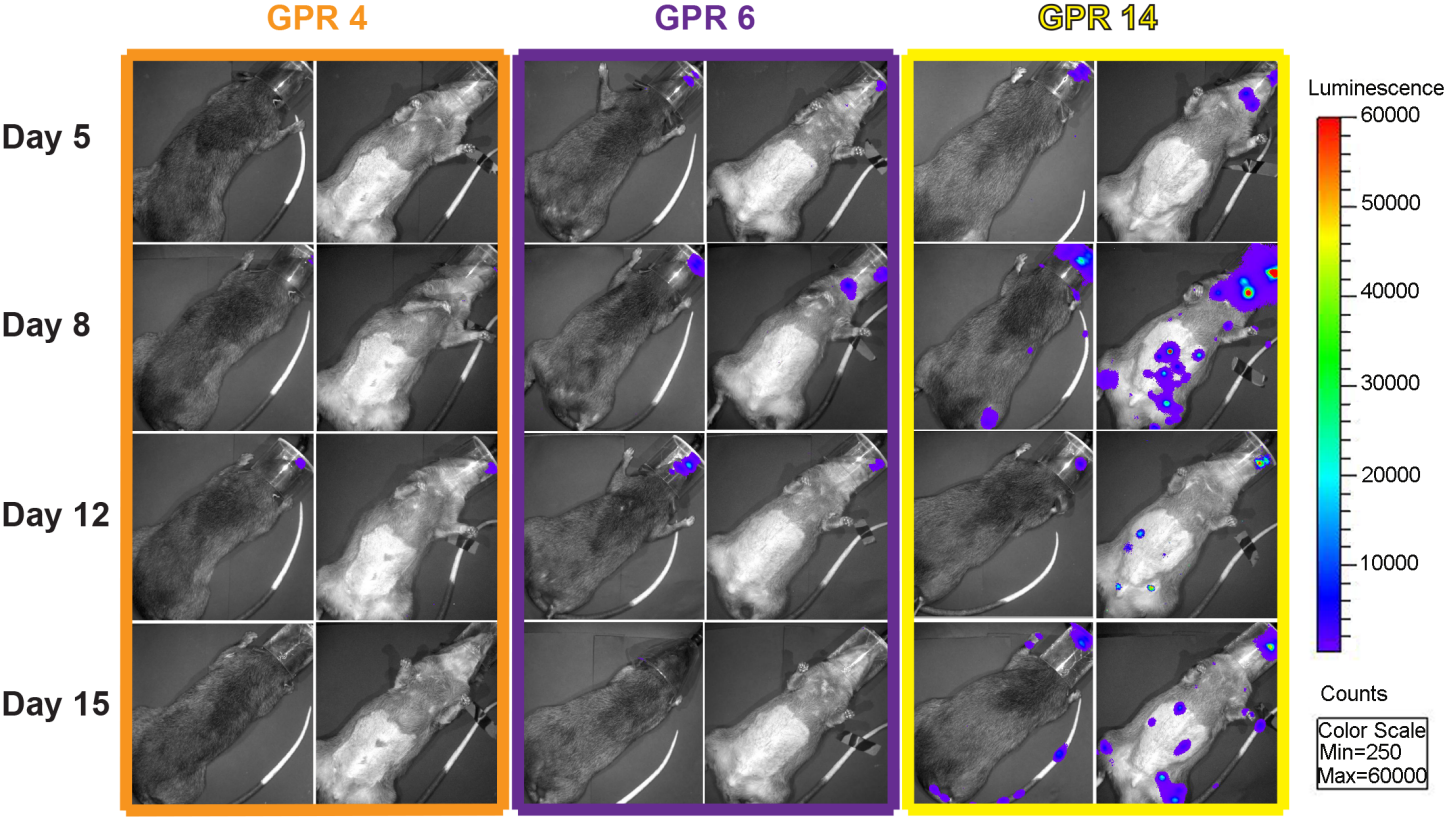
Dorsal and ventral views of Gambian pouched rats (*Cricetomys gambianus*) infected intranasally with MPXV/Luc+. Gambian pouched rats infected intranasally with *Monkeypox virus* that expresses firefly luciferase (MPXV/Luc+) show luminescence (viral replication) at the initial site of inoculation, in the nasal area. In two animals (GPR 6 and GPR 14), there is luminescence in the region of the submandibular lymph nodes, a common site of secondary replication for Orthopoxviruses. In GPR 14, luminescence is evident in distant sites from day 8 to day 22 post-infection. Despite evidence of viral replication, none of these animals showed any grossly visible skin lesions.

**Fig 3:**
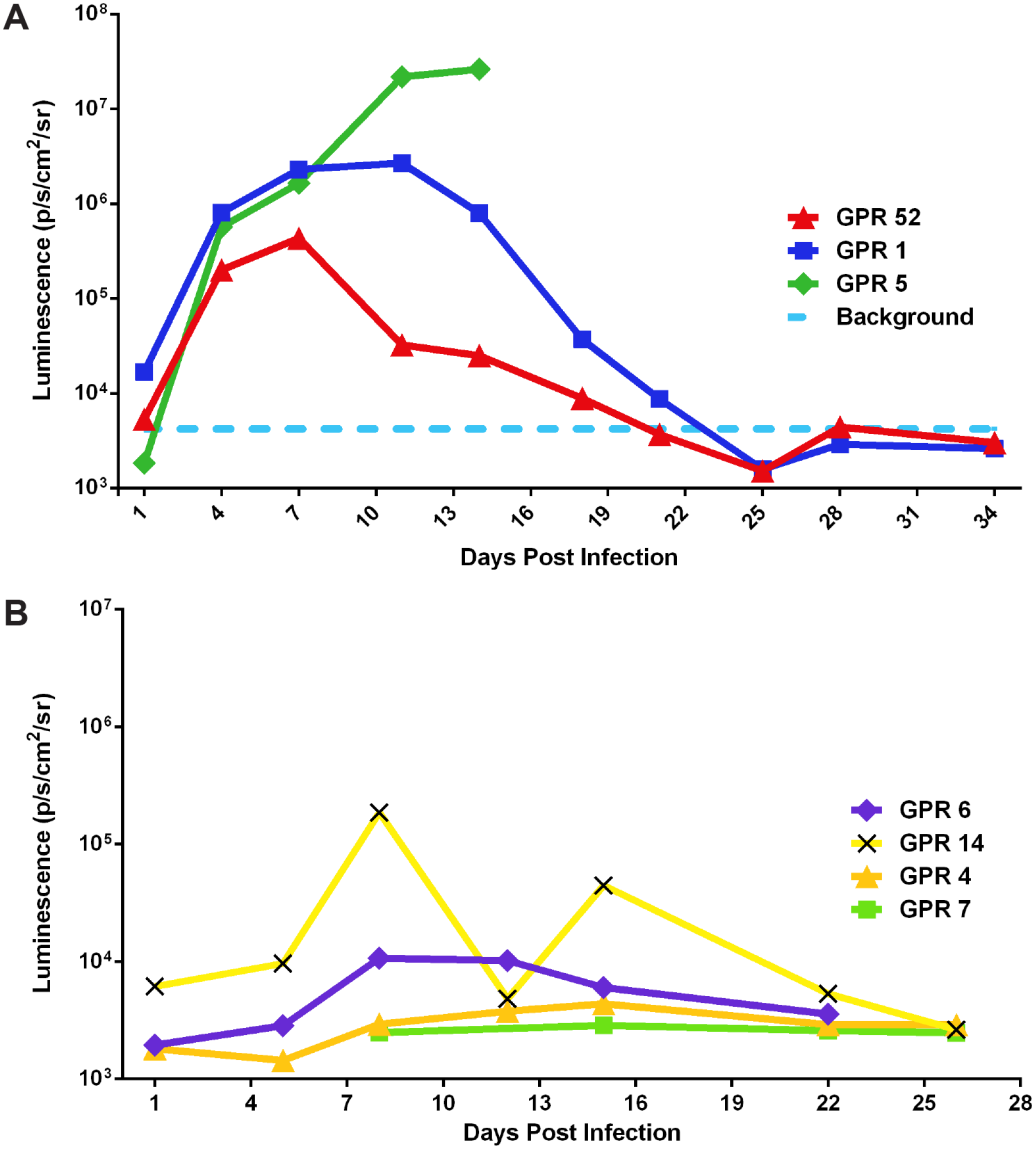
Luminescence of Gambian Pouched Rats (*Cricetomys gambianus*) infected with Monkeypox/Luc+ by intradermal (A) and intranasal (B) routes. Total luminescence (p/s/cm^3^/str), an estimate of viral load, peaks in all animals between days 8 and 14 and returned to background levels by day 21 presumably in relation to viral load. The total luminescence of the negative control (GPR 51), taken at day 0, is shown as a dashed line (A), to estimate background luminescence of Gambian pouched rats. The total luminescence of the sentinel animal (GPR 7) is shown in B. It remained similar to that of the negative control.

After infection with either an ID or IN inoculation of 10^6^ pfu of MPXV/ Luc+, BLI revealed luminescence, indicative of viral replication, at the primary sites of inoculation, followed by spread to secondary and tertiary tissues. In ID infected animals (GPR 1, GPR 5, GPR 52), luminescence was detected at the site of infection 3 dpi (Fig 1). By 7 dpi, luminescence was detected in the eyes, nose, mouth, and in many areas of the skin (Fig 1). It was also detected at distant sites of the skin, as well as the nose, by 7 dpi (Fig 1). In IN infected animals (GPR 4, GPR 6, GPR 14), luminescence was initially detected in the nasal cavity, and then in the mouth, at the presumed sites of the superficial cervical lymph nodes and in distant sites of the skin (Fig 2). Total luminescence peaked in all infected animals between 8 and 14 dpi (Fig 3) and returned to background levels in all animals by 21 dpi. In one animal, GPR 14, luminescence unexpectedly decreased on 12 dpi. We believe this was caused by an inadvertent injection of the luciferin substrate into the intestine or bladder, instead of the peritoneum, that resulted in decreased distribution of the substrate and decreased luminescence. The total luminescence of the negative control (GPR 51) was 4.21 x 10^3^ p/s/cm^3^/str, prior to mock-infection, and the luminescence of the sentinel remained close to levels of the negative control throughout the study (2.47–2.87 x 10^3^ p/s/cm^3^/str) (Fig 3A and 3B, respectively). All luminescence measurements are listed in S1 Table.

### Clinical Signs

**Fig 4:**
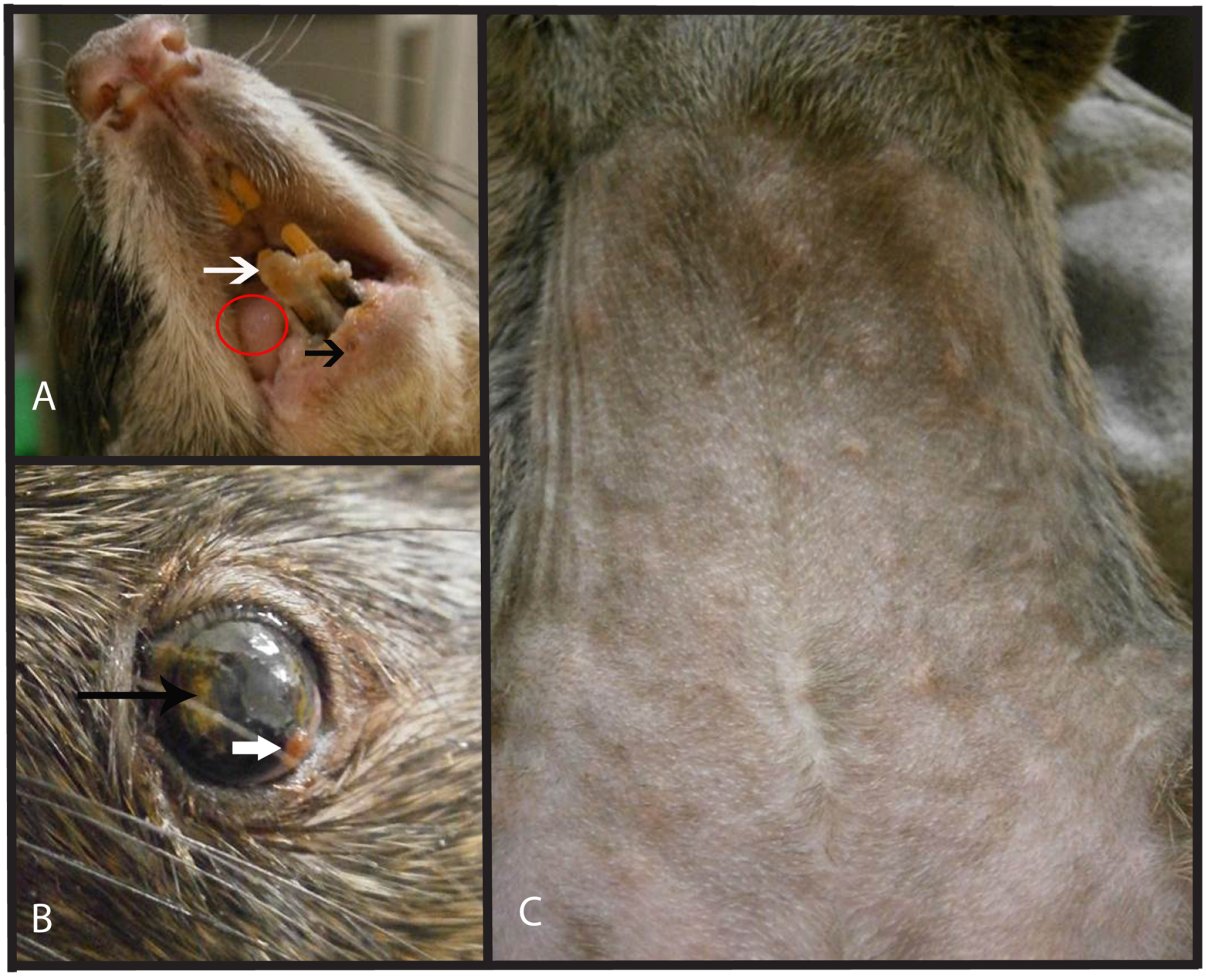
Gross lesions of Gambian pouched rats (Cricetomys gambianus) infected with MPXV/Luc+. A. GPR 5, 14 days after infection with *Monkeypox Virus* expressing luciferase (MPXV/Luc+) with a vesicle on the tongue (red circle), necrotic gingivitis (white arrow) and a vesicle that has opened and crusted (black arrow). B. The left eye of GPR 1 with white-yellow corneal opacities (black arrow) and ocular discharge (white arrow). C The abdomen of GPR 5, 14 days after infection, with prominent ribs and folds of skin which show severe weight loss, as well as many skin vesicles on the abdomen. Luminescence (fig 3) is visible in all of these areas and there were high levels of viral shedding in the conjunctival/corneal swabs taken from GPR 1, as well as in oral swabs taken from GPR 5.

**Fig 5:**
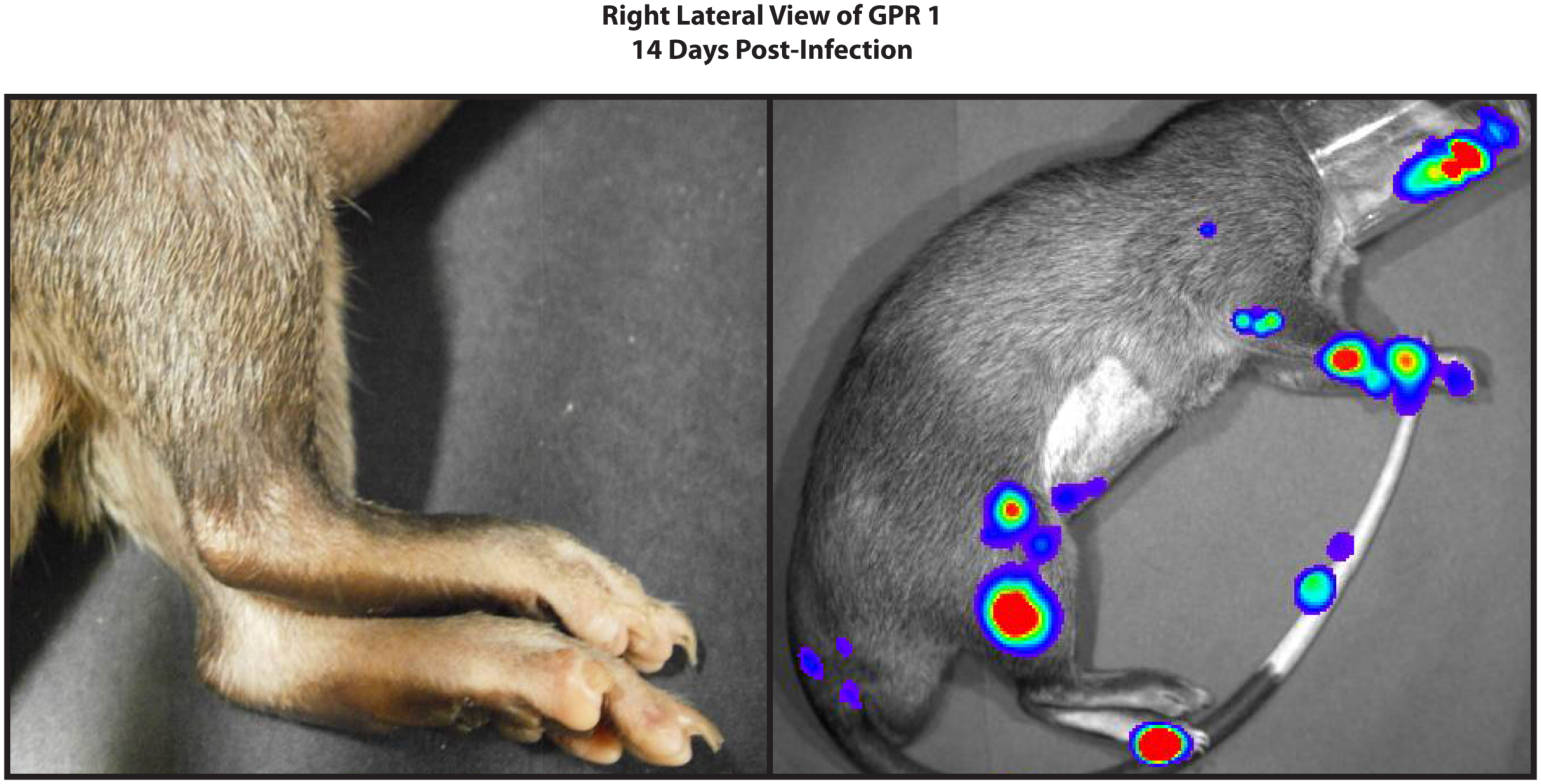
Luminescence is present in the absence of gross lesions of monkeypox disease. In this photograph (left) of the right lateral leg of GPR1, 14 days after it was infected with *Monkeypox virus* that expresses luciferase, no skin lesions are apparent. However, on the right, the luminescent image of the same animal, on the same day, shows abundant viral replication in the leg and foot of this animal. This is clear evidence that viral replication can occur in the absence of visible skin lesions.

Intradermal infection of Gambian pouched rats resulted in three distinct clinical outcomes. One animal (GPR5) became clinically ill, displaying lethargy, anorexia, 17% weight loss, numerous skin lesions, vesicles on the tongue, and necrosis of the gingiva (Fig 4). This animal was euthanized on 17 dpi, having met the euthanasia criteria of difficulty eating and severe lethargy. A second animal (GPR1) became clinically ill, but recovered by 18 dpi. This animal had secondary skin lesions, lethargy, anorexia, 5.4% weight loss, and ocular lesions, but recovered and was clinically normal by 24 dpi, with the exception of a small scab still present at the site of inoculation. Although skin lesions were observed in this animal, there was also evidence of viral replication in some areas of the skin despite the absence of visible lesions at those sites (Fig 5). A third animal (GPR 52) displayed only a primary lesion at the site of ID inoculation. This animal displayed no other clinical signs, despite evidence of active viral replication via BLI (Fig 1). Intranasally infected animals (GPR 4, GPR 6, GPR 14) developed no visible skin or oral lesions, despite evidence of viral replication via BLI in these regions (Fig 2). Likewise, the sentinel and negative control animals did not display any signs of disease.

### PCR and Viral Isolation from Tissues

PCR and cell culture results from tissues of the ID infected group are shown in Table 1. Briefly, many tissues from GPR5, the animal euthanized at 17 dpi, were positive by real time PCR. Kidney from GPR52 was MPXV-positive by PCR, although viral replication was not detected via BLI in the region of the kidney, nor was the tissue positive for viable virus by the TCID_50_ assay. Spleen and the primary skin lesion of the recovered animal, GPR1, were still PCR-positive at the date of euthanasia (34 dpi). No live virus was detected in tissues from IN infected animals. Lung, spleen and esophagus of GPR 14 were PCR-positive, as well as the kidney of GPR 6. The mostly negative results in the IN infected group are not surprising, given that tissues were collected at the end of the study, when BLI indicated that virus was no longer actively replicating or was replicating below the level of detection. No viral DNA was detected in either the control or sentinel tissues.

**Table 1:** PCR and Viral Titration of Tissues from Gambian Pouched Rats Infected ID and IN with Monkeypox/Luc±.

	Intradermal inoculation						Intranasal inoculation					
	GPR 1		GPR 5		GPR 52		GPR 6		GPR 4		GPR 14	
Tissue	PCR	PFU/ml	PCR	PFU/ml	PCR	PFU/ml	PCR	PFU/ml	PCR	PFU/ml	PCR	PFU/ml
Salivary Gland	-	0	+	6.90E+07	-	0	-	0	-	0	-	0
Submandibular Lymph Node	-	0	-	2.18E+02	-	0	-	0	-	0	-	0
Heart	-	0	+	0	-	0	-	0	-	0	-	0
Liver	-	0	+	0	-	0	-	0	-	0	-	0
Lung	-	0	+	1.77E+07	-	0	-	0	-	0	+	0
Spleen	+	0	+	4.35E+04	-	0	-	0	-	0	+	0
Intestine	-	0	+	1.09E+07	-	0	-	0	-	0	-	0
Stomach	-	0	NT	0.00E+00	-	0	-	0	-	0	-	0
Kidney	-	0	+	1.77E+02	+	0	+	0	-	0	-	0
Testis/Ovary	-	0	+	4.42E+04	-	0	-	0	-	0	-	0
Brain	-	0	+	4.35E+05	-	0	-	0	-	0	-	0
Skin	+	3.20E+04	+	1.34E+07	-	0	-	0	-	0	-	0
Tongue	-	0	+	1.49E+06	-	0	-	0	-	0	-	0
Bladder	-	-	-	-	-	-	-	0	-	0	-	0
Inguinal Lymph Node	NT	NT	+	2.18E+07	NT	NT	NT	NT	NT	NT	NT	NT
Esophagus	NT	NT	NT	NT	NT	NT	NT	NT	NT	NT	+	NT
Blood/Viremia	-	NT	-	NT	-		-	NT	-	NT	-	NT
	(D34)		(D34)		(D28)		(D27)		(D27)		(D26)	

*NT = Not tested or not collected

+ positive

- negative

Six Gambian pouched rats (*Cricetomys gambianus*) were infected intradermally (GPR 1, GPR 5, GP52) or intranasally (GPR 4, GPR 6, GPR 14) with 10^6^ PFUs of *Monkeypox virus* expressing firefly luciferase. Viral titration and PCR results show that many tissues are infected during active disease (GPR 5), but few tissues remained infected after resolution of clinical signs (GPR 1, GPR 52, GPR 6, GPR 14). Viremia (by PCR) is shown for the last day on which a whole blood sample was collected prior to euthanasia and the day of collection is indicated in parentheses. 10% (wgt/vol) homogenates were made from tissues and viral titrations are reported as PFU/ml of slurry. Viral titration was by TCID_50_ method, using Vero cell culture. PCR was performed using a generic *Orthopoxvirus* E9L Q-PCR assay. All tissues from the negative control (GPR 51) and sentinel (GPR 7) were negative by PCR and TCID_50_.

### Viral Shedding and Viremia

**Fig 6:**
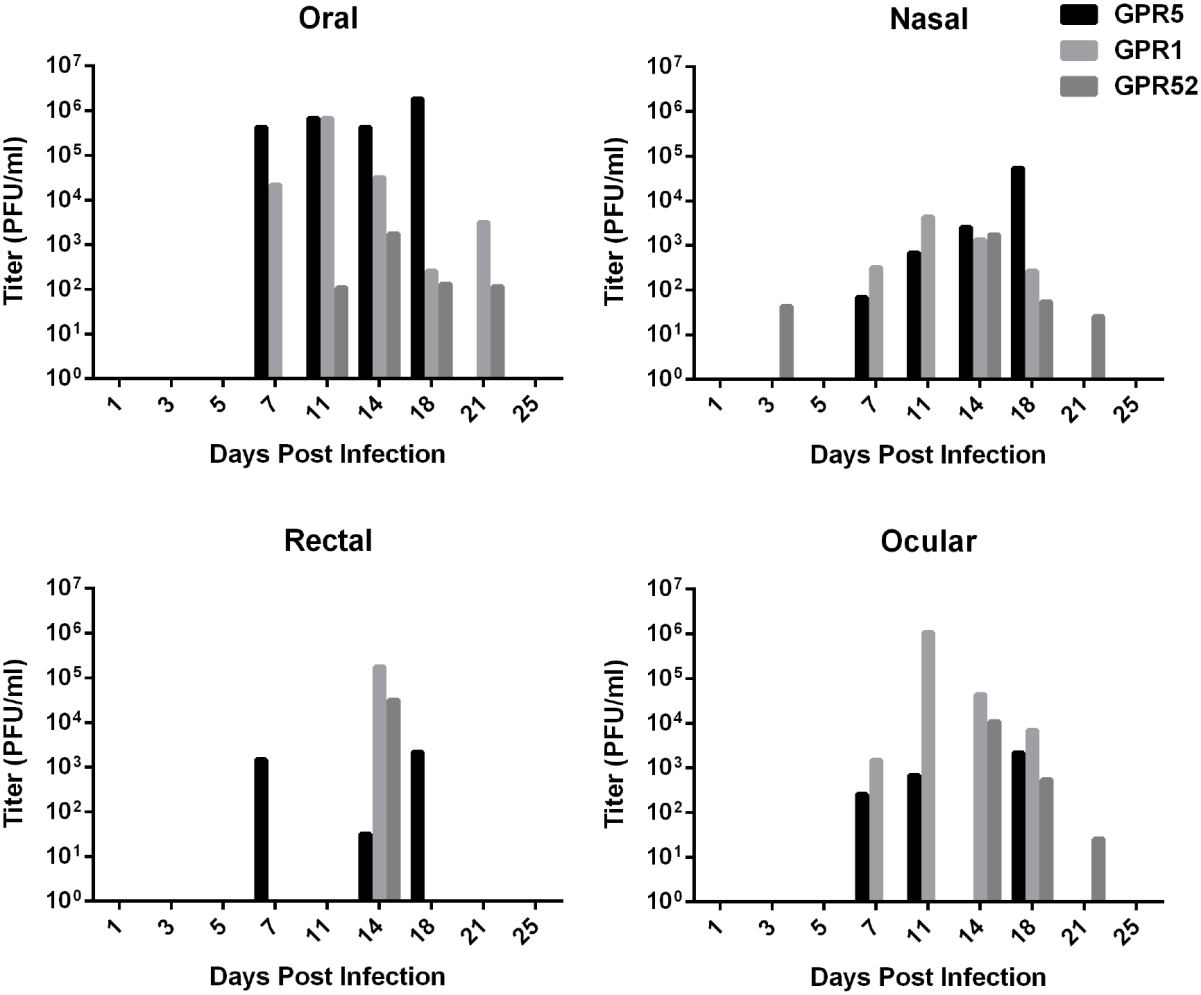
Shedding of *Monkeypox virus* in oral, nasal, rectal, and ocular swabs from intradermally infected Gambian pouched rats (*Cricetomys gambianus*). Viral shedding was detected by TCID50 assay from swabs of oral, nasal, rectal, and ocular mucosal surfaces. Oral shedding was as high as 10^6^ pfu, which was the dose used to infect the animals. All three infected animals shed in oral, nasal, rectal, and ocular swabs.

**Fig 7:**
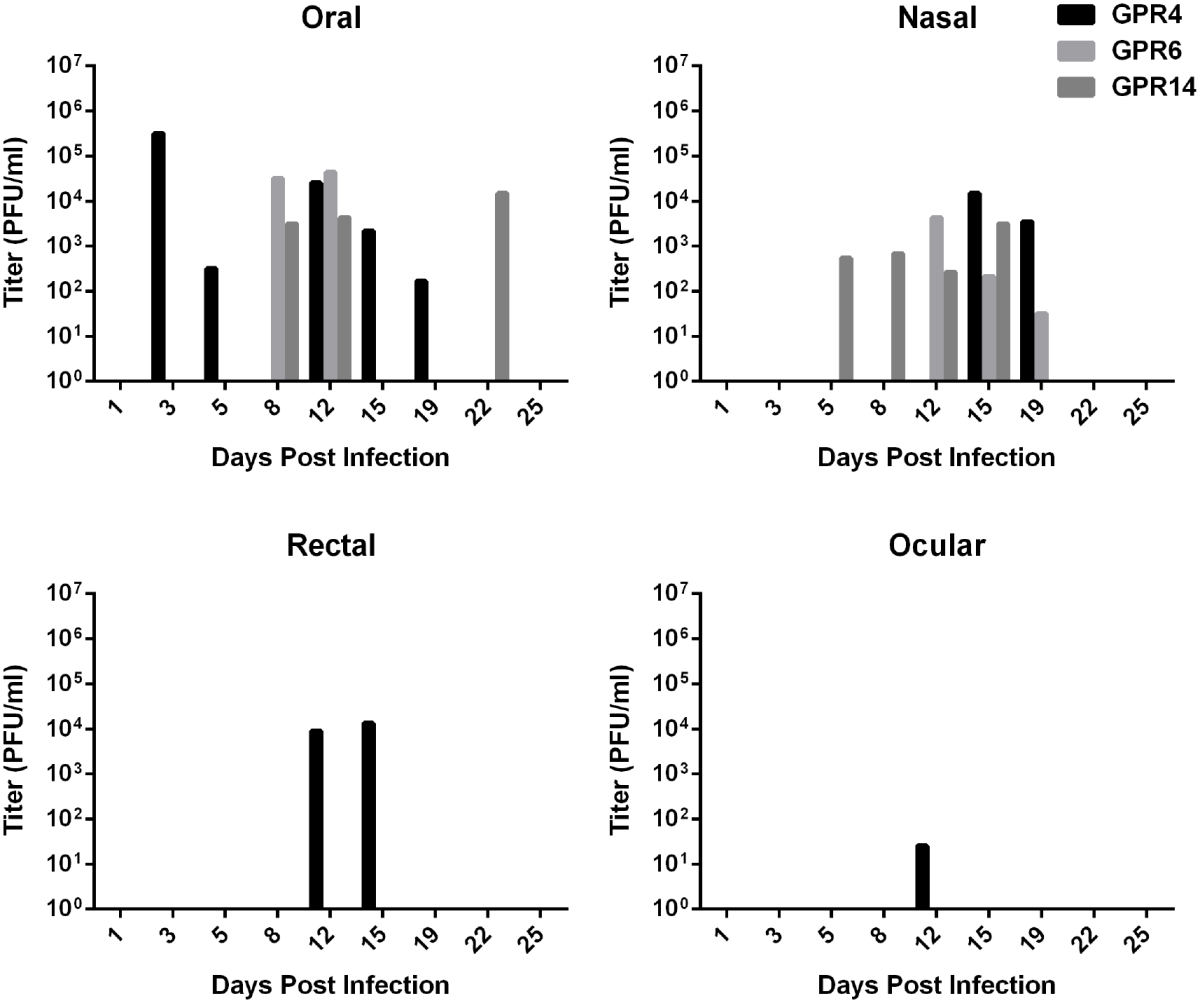
Shedding of *Monkeypox virus* in oral, nasal, rectal and ocular swabs from intranasally infected Gambian pouched rats (*Cricetomys gambianus*). Viral shedding was detected by TCID50 assay from swabs of oral, nasal, rectal, and ocular mucosal surfaces. Shedding began slightly earlier than in intradermally infected rats (fig 8), but was lower in titer.

Viral shedding was detected by TCID_50_ cell culture assay from the media in which oral, rectal, ocular, and nasal swabs were frozen. No viral DNA was detected via PCR in urine. Viremia was detected by q-PCR in one IN infected animal (GPR4) on 5 dpi and one ID infected animal (GPR 1) on 11 dpi. Shedding of MPXV occurred in oral secretions of all infected animals, with titers of oral swabs ranging from less than 100 pfu to 1.85 x 10^6^ pfu/ml. Each animal also shed by at least one other route, although the pattern of shedding varied between animals (Figs 6 and 7). Swabs were collected from GPR 5 just before euthanasia on 17 dpi, and these are reported in the figures as 18 dpi to simplify the graphics. Oral, rectal, and nasal shedding were not significantly different between the ID and IN infected groups (p = 0.1135, p = 0.3893, p = 0.5149, respectively). Ocular swabs from all 3 animals in the ID infected group were positive for virus, but only 1 ocular swab was positive in the IN infected group. Thus mean titers were not compared between groups. None of the swabs from the sentinel or negative control were positive for live virus by TCID_50_.

### Anti-orthopox Antibodies

**Fig 8:**
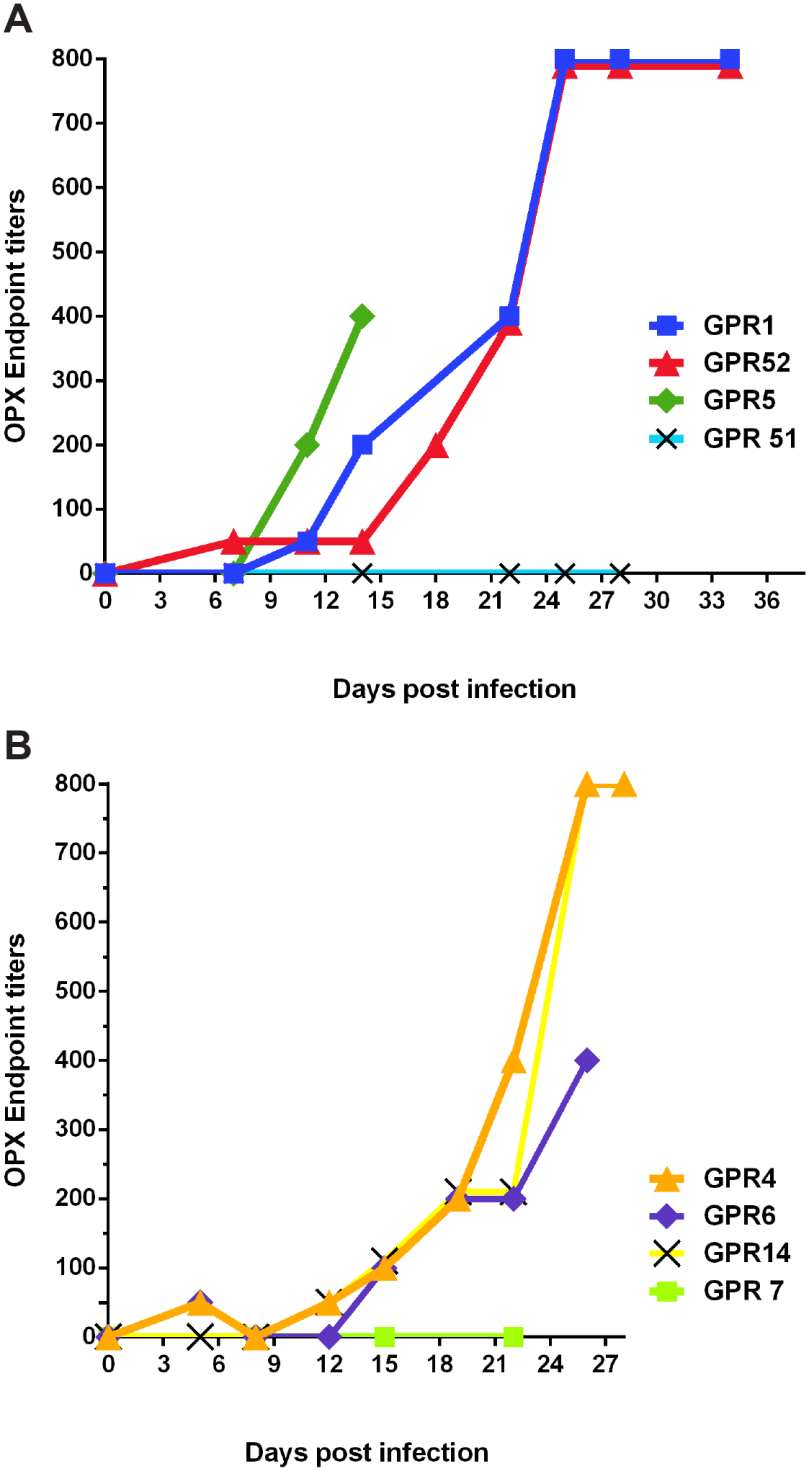
Anti-Orthopox virus antibody titers of animals infected intradermally (A) and intranasally (B) with *Monkeypox virus* expressing luciferase (MPXV/Luc+). Antibody titers indicate that animals develop a strong humoral immune response around 3 weeks, which coincides with the time at which luminescence and viral shedding begins to cease. Orthopox antibodies are strongly cross-reactive, so antibody responses to *Vaccinia virus*, as in this method, very closely approximate titers to *Monkeypox virus*.

All animals were sero-negative before the study. Antibody titers began to rise between 11–12 dpi (Fig 8). By the end of the study at 26 to 34 dpi, all surviving infected animals were seropositive, with titers ranging from 1:400 to 1:800. The negative control (GPR 51) and the sentinel (GPR 7) remained sero-negative throughout the duration of the study (S2 Table).

## Discussion

Most notably, this study demonstrated that Gambian pouched rats can be infected with and shed MPXV in the absence of clinical signs of disease. Infected Gambian pouched rats shed up to 10^6^ pfu of virus, which is an infectious dose for many species, including non-human primates and other rodents [36,37,38]. The ID route of infection was more pathogenic than the IN route, although both routes resulted in shedding of live virus. Clinical characteristics of MPXV infection and shedding provide valuable information as to the potential role of this rodent species as a reservoir for MPXV. The ability to survive infection for a reasonable time period is an important trait for a reservoir host, as it must be able to transmit the virus to other individuals in order to maintain viral circulation. This study did not find evidence of aerosol transmission between Gambian pouched rats when animals were housed in individual cages within the same room. The sentinel animal was seronegative at the end of the study and no virus was detected in tissues, blood, or swabs from this animal. Direct contact or fomite transmission may be required. Pouched rats are solitary animals that live in burrows [39,40]. To easily transmit the virus they must be healthy enough to leave the burrow and come into contact with other animals or shed the virus into the environment. Our data indicate that an infected Gambian pouched rat is able to shed virus in oral, nasal, ocular, and rectal secretions, and most infected animals remain healthy enough to maintain normal foraging behavior. Viral titers in oral secretions were greater than 10^6^ pfu, which has now been demonstrated to be an infectious dose for Gambian pouched rats. From other studies, we know this is also a lethal dose for cynomologous macaques, prairie dogs, and ground squirrels [36,37,38]. Thus, an infected pouched rat could transmit to other con-specifics via fomites, such as a shared food source, or via direct contact during mating or fighting.

The results reported herein complement a parallel study conducted by collaborators with Gambian pouched rats infected at a lower challenge dose (Hutson et. al. in press). In that study, rats were challenged ID with either the parental Congo basin MPXV strain that was used in our study or with a West African MPXV strain (both at 4X10^4pfu). With the exception of one animal that died in the West African MXPV group, all other animals developed cutaneous skin lesions, shed high loads of virus from multiple swab samples throughout the course of the study (lesion, nasal, oral, rectal) and recovered from infection. Telemetry monitoring was used to record the activity level of each rat, and although animals developed cutaneous lesions and were shedding virus, activity of challenged rats were not significantly different than control animals when compared on a day to day basis (although there were differences in average activity levels between the groups). This data confirms our findings that Gambian pouched rats infected with MXPV do not become moribund while shedding virus and, therefore, may actively transmit the virus to both animals and people in natural settings.

In this study, we used a recombinant MPXV expressing luciferase (MPXV/Luc+). Previous studies showed that in CAST/EiJ, SCID Balb/C and Balb/C mice, virulence was not affected by the insertion of the luciferase gene [24,27]. This study did not directly compare wild type virus to recombinant virus in Gambian pouched rats. However, the parallel study in Gambian pouched rats performed by Hutson et. al. also suggests that Gambian pouched rats do not become severely ill from the parental type. Thus, it is unlikely that the observed pathogenicity was affected by the insertion of the luciferase gene.

Our results also suggest possible routes of transmission of MPXV from Gambian pouched rats to humans. BLI demonstrated apparently superficial viral replication in the absence of skin lesions. Although the luminescence often had the characteristic “bull’s eye” appearance seen in visible pustular lesions, such lesions were not always visible to the naked eye. BLI did not conclusively locate this viral replication to the skin; it may have been present in the epidermis, dermis, or subcutaneous lymph nodes. In any case, this data raises the possibility that humans may come into contact with MPXV through hunting and butchering of an apparently healthy pouched rat. This is, in fact, a distinct possibility in DRC and other regions of Africa, where the pouched rat is hunted for food [18]. A second conceivable source of infection for humans would be through fomites, such as food items, which may be scavenged by pouched rats in the fields or in homes, and then contaminated with MPXV. This type of indirect contact is thought to be responsible for much of the rodent-to-human transmission of Lassa fever virus in West Africa [41].

Although this study demonstrates that the Gambian pouched rat has characteristics that would make it a good reservoir host, it is not the only candidate on the list of potential reservoirs. Studies conducted since the 1980s have implicated a number of species as potential reservoirs. In 1985, MPXV was isolated from a moribund rope squirrel in Zaire, now DRC [42]. Serological studies in the 1980s in Zaire suggested that rope squirrels (*Funisciurus anerythrus*) and sun squirrels (*Heliosciurus rufobrachium*) were important as reservoirs for MPXV [43]. In Ghana, serological and PCR assays suggest the presence of orthopoxviruses in Gambian pouched rats, rope squirrels, sun squirrels, dormice (*Graphiurus spp*.) and African ground squirrels (*Xerus spp*.) [6]. Following the accidental importation of MPXV into the United States from Ghana in 2003, Gambian pouched rats, rope squirrels and dormice were all found to be positive for MPXV DNA and live MPXV [15]. Most recently, MPXV was isolated from a dead sooty mangabey found in (*Cercocebus atys*) in Côte d’Ivoire [10], although it is unclear whether this species represents an additional potential maintenance host or only a spillover host, like humans.

Recently, investigators used remote sensing to compare the distribution of human MPX cases in relation to habitat of possible reservoir hosts and concluded that the habitat of arboreal rope squirrels correlated most strongly with the reported human cases. [44]. The combined results of all of these studies could suggest that MPXV is not maintained by one host in the wild, but rather can be maintained by several rodent species. Further study is needed to determine the roles that each of these species plays in maintenance, transmission, and amplification of the virus.

BLI is an effective tool for tracking replicating MPXV in live animals. In previous studies, we have shown that in large rodents, such as prairie dogs, some of the luminescent signal in deep tissues is attenuated by overlying tissues, reducing sensitivity in comparison with small rodents such as mice [25]. However, BLI was effective in targeting specific organs for later virus isolation and for estimating the number of viral lesions in the skin; it could also identify viral replication in the absence of lesions. This is especially important for identifying possible reservoir hosts that may spread the virus with little or no clinical signs.

In this study, BLI revealed replication of MPXV in both healthy and sick animals. Although we were unable to correlate luminescence to an exact titer in different tissues, we can track the relative amount of virus in the animals over time. In both ID and IN infected animals, viral replication peaked between 8 and 14 dpi. Using BLI, we also demonstrated that the virus returned to background levels by 21 dpi, indicating that neither ID nor IN inoculation result in the establishment of chronic infection.

Viral culture results confirmed the absence of live virus in tissues from most animals at the end of the study (Table 1). Live virus was detected in the skin and associated scab at the site of inoculation of GPR 1. Active viral replication (and thus, luciferin expression) may have ceased at this point in the infection. Orthopoxviruses are known to survive for long periods in scabs or tissue crusts after active infection is resolved [45,46]. Several tissues were PCR positive, despite being negative by viral culture (GPR 5 liver, GPR 5 heart, GPR 52 kidney, GPR 6 kidney, GPR 14 lung, GPR 14 spleen). These results likely reflect DNA present in tissues at sites of previous infection. One tissue, GPR 5 submandibular lymph node, was negative by PCR, but live virus was detected by TCID50 assay (218 pfu/ml). The DNA sample from this tissue may have contained PCR inhibitors, or the level of viral DNA present may have been below the limit of detection of the real time PCR assay. Unfortunately, the sample volume was very small and none remained to repeat the DNA extraction.

The limits of detection of the real time PCR assay may have also reduced the ability to detect viremia prior to spread to secondary sites of replication. Nasal and oral swabs were positive by viral culture on 3 dpi from GPR 1 and GPR 4, respectively. However, viremia was not detected until 5 dpi in GPR 4 and 11 dpi in GPR 1. Viremia was not detected in other rats. In orthopoxvirus and other poxvirus infections, a cell-associated viremia develops after initial inoculation [47,48,49]. Therefore, a more sensitive sample for detecting viremia would have been peripheral blood mononuclear cells (PBMCs). However, the facility in which anesthesia and imaging took place in this study did not have the proper equipment needed to immediately process blood samples under BSL3 conditions. Therefore, we were unable to collect PBMCs, which limited our ability to detect viremia in this study.

It is also interesting to note that nasal swabs were not immediately positive for live virus 1 day after intranasal infection. Likewise, there was no large increase in luminescence in the oronasal region until 5 dpi. Local replication at the initial site of infection may have been low or established at a location within the nasal passages more distal than where the nasal swab could reach. Live virus detected in nasal swabs at 5 dpi likely represents shedding after viremia and spread to secondary sites of infection, rather than local infection only. Unfortunately, the inability to detect viremia in many of the rats somewhat limits our ability to verify this series of events.

In conclusion, this study demonstrated that pouched rats should be considered a potential source of MPXV infection for humans, as healthy pouched rats may shed virus for several weeks. Continued study is needed to determine a 50% infectious dose and shedding patterns by other routes of infection. Furthermore, ecological and behavioral studies should be conducted to assess the likelihood of pouched rats serving as a reservoir host, or alternatively as a transmission host of MPXV to humans, with another species serving to maintain the virus in nature.

## Supporting Information

S1 Table Luminescence data from dorsal and ventral views of Gambian pouched rats (*Cricetomys gambianus*) infected with Monkeypox/Luc+. Luminescence (p/s/cm^3^/str) was calculated from free-drawn regions of interest (ROIs) and analyzed with Living Image version 4.2 software. This data was used to calculate total luminescence, as seen in fig 3.
(XLSX)

S2 Table Optical Densities and end-point titers of anti-Orthopox ELISAs. Optical densities measured during enzyme linked immunosorbent assays (ELISAs) from sera of infected and uninfected Gambian pouched rats (*Cricetomys gambianus*). Cutoffs for each plate were calculated based on optical densities measured in response to uninfected Vero cell lysate. End -point titers in this table were used to construct fig 8.
(XLSX)
